# Supplementary material for: The ecological and developmental foundations of brood parasitism in a catfish
Source: Nat Commun. 2026 Mar 31;17:4630. doi: 10.1038/s41467-026-71179-4 (PMC13199399; doi:10.1038/s41467-026-71179-4)

Supplementary Information for:

The ecological and developmental foundations of brood parasitism in a catfish

Martin Reichard[1,2,3,*], Radim Blažek[1,3], Matej Polačik[1], Tomáš Suchánek[4], Gernot K. Englmaier[1], Kacper Pyrzanowski[1,2], Veronika Bartáková[1], Jakub Žák[3], Lukas Koch[1,3], Iva Dyková[3], Robert Cerny[4], Stephan Koblmüller[5], Holger Zimmermann[5,1,*]

[1] Institute of Vertebrate Biology, Czech Academy of Sciences, Květná 8, Brno, Czech Republic
[2] Department of Ecology and Vertebrate Zoology, University of Lodz, Banacha 12/16, 90-237 Lodz, Poland
[3] Department of Botany and Zoology, Faculty of Science, Kotlářská 2, Masaryk University, Brno, Czech Republic
[4] Department of Zoology, Faculty of Science, Charles University in Prague, Czech Republic
[5] Institute of Biology, University of Graz, Universitätsplatz 2, 8010 Graz, Austria

*Correspondence: reichard@ivb.cz (MR), holger.zimmermann@uni-graz.at (HZ)

Figures S1-S4

Table S1-S13

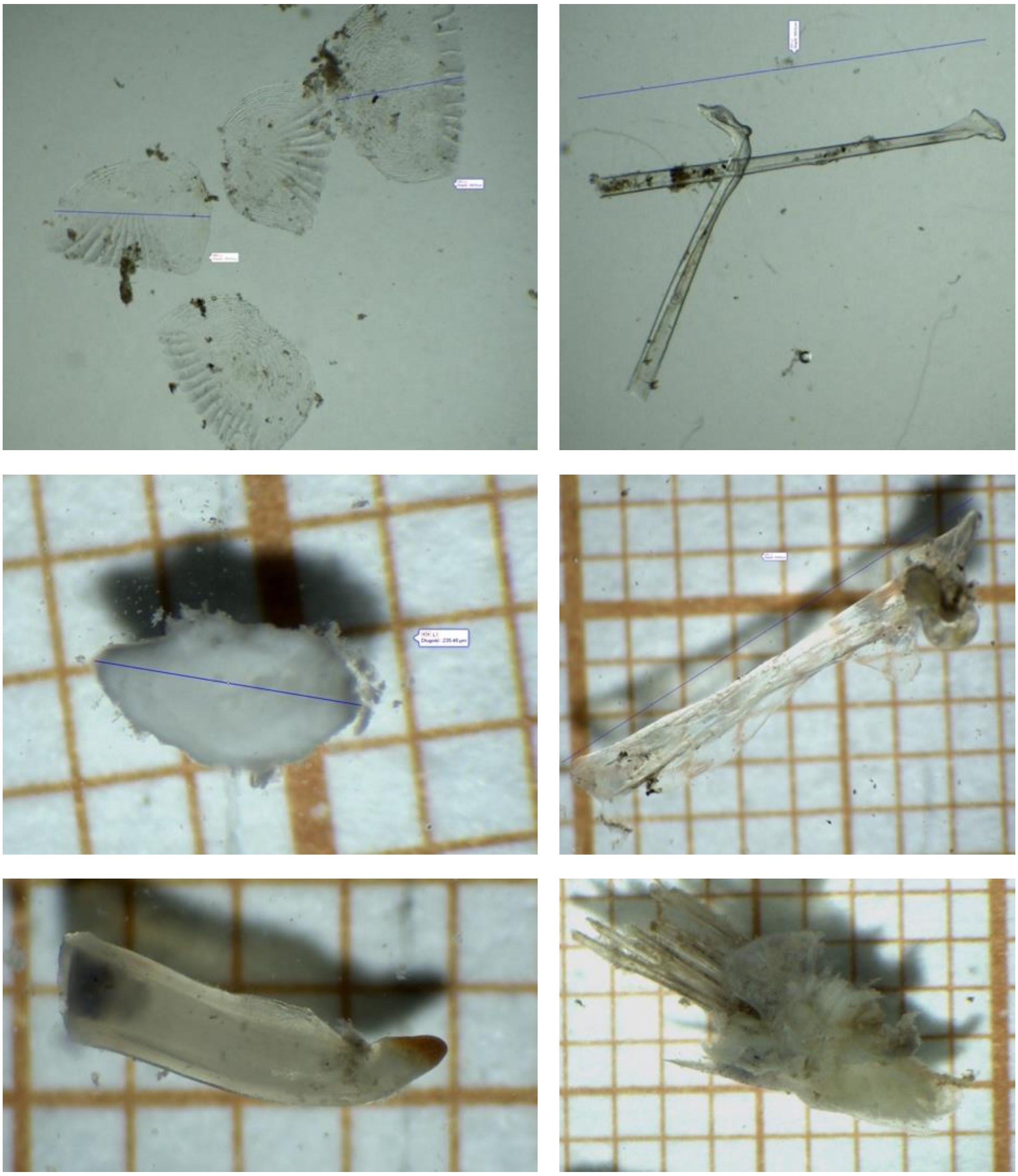

Figure S1 | An example of *S. granulosus* diet composed of fish scales, bones, otoliths and tooth.

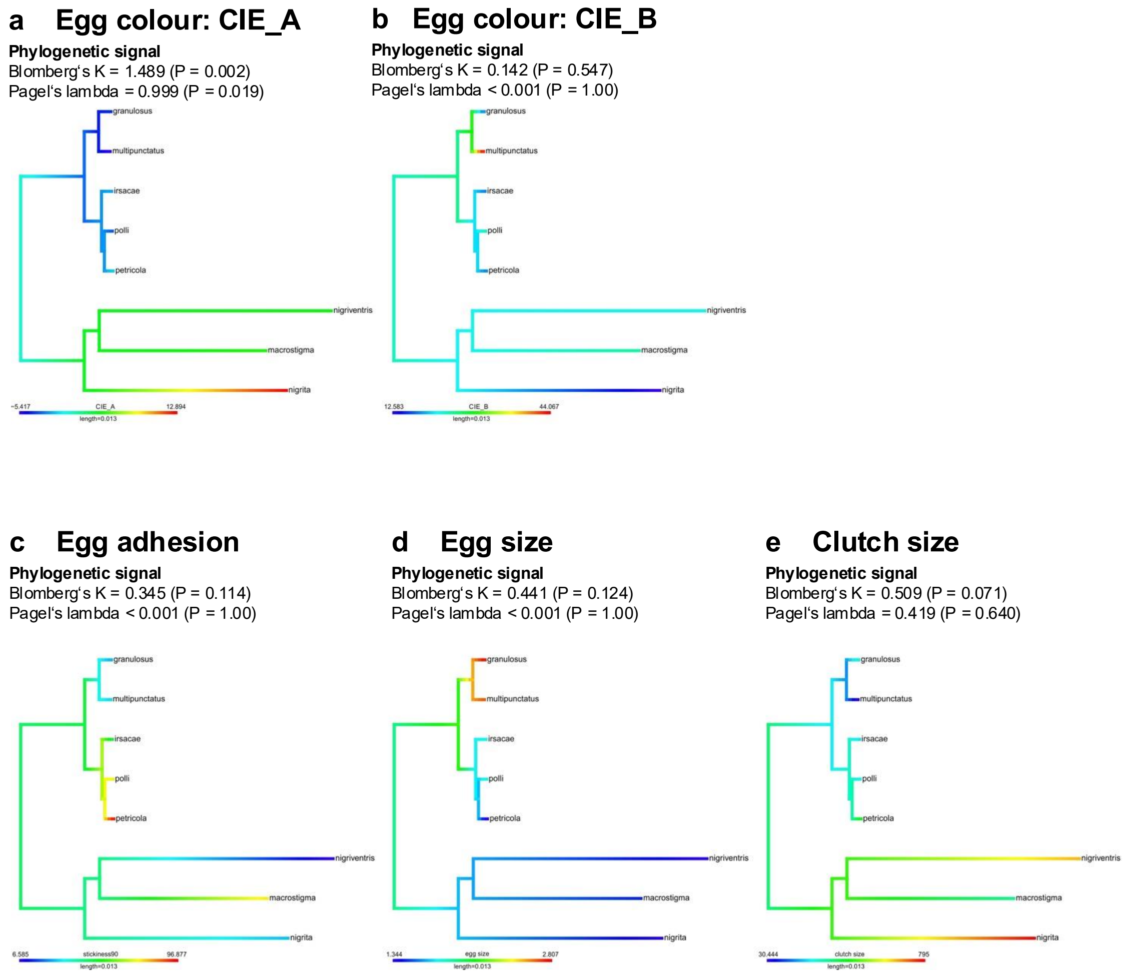

Figure S2 | Phylogenetic contrasts. Ancestral trait reconstruction and phylogenetic signals of reproductive traits in five LT and three riverine *Synodontis*. Traits were modelled as continuous characters using the *fastAnc* function in *phytools*.

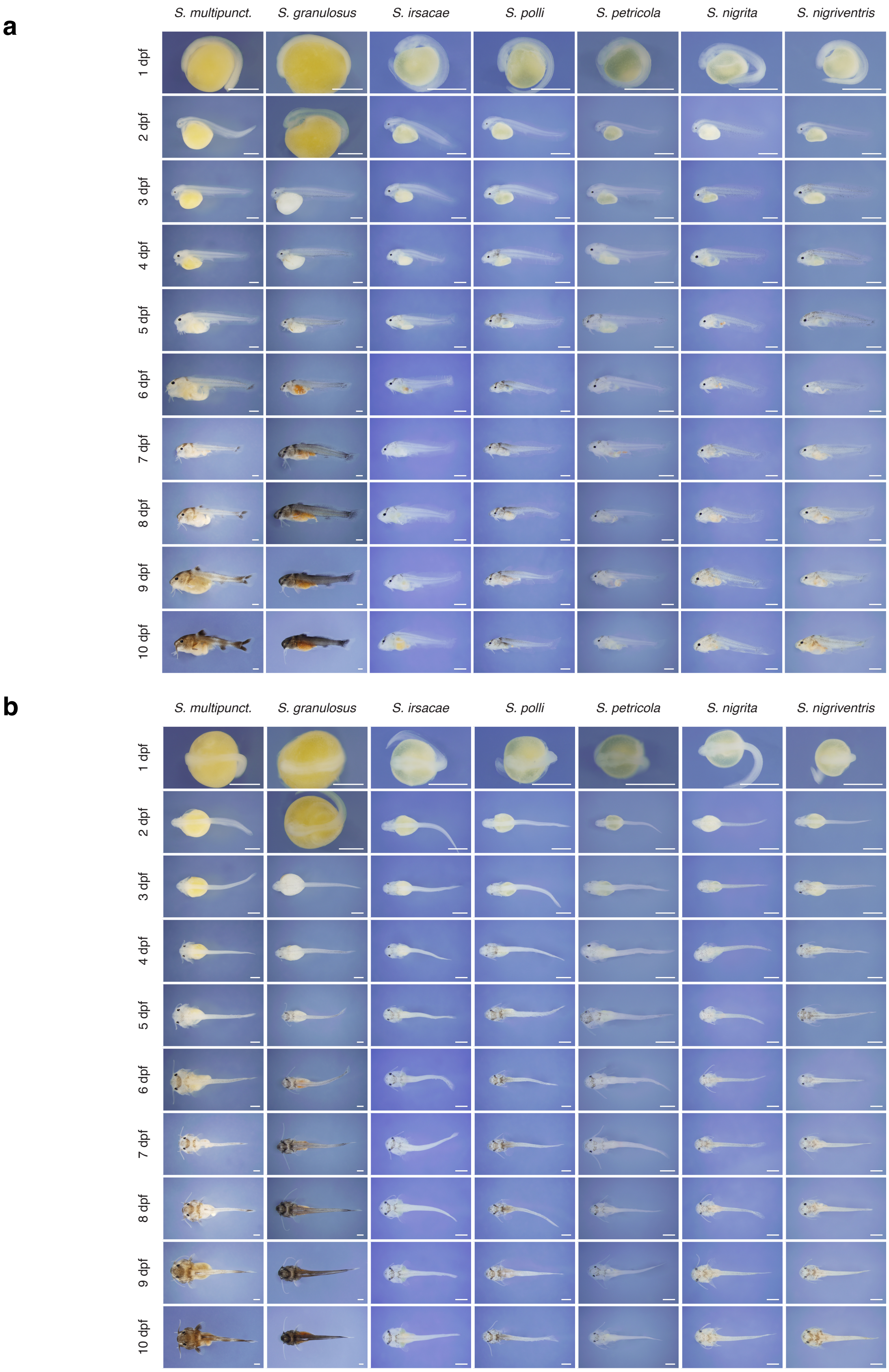

Figure S3 | Lateral (**A**) and dorsal (**B**) views of *Synodontis* embryo development. Scale: bar = 1 mm.

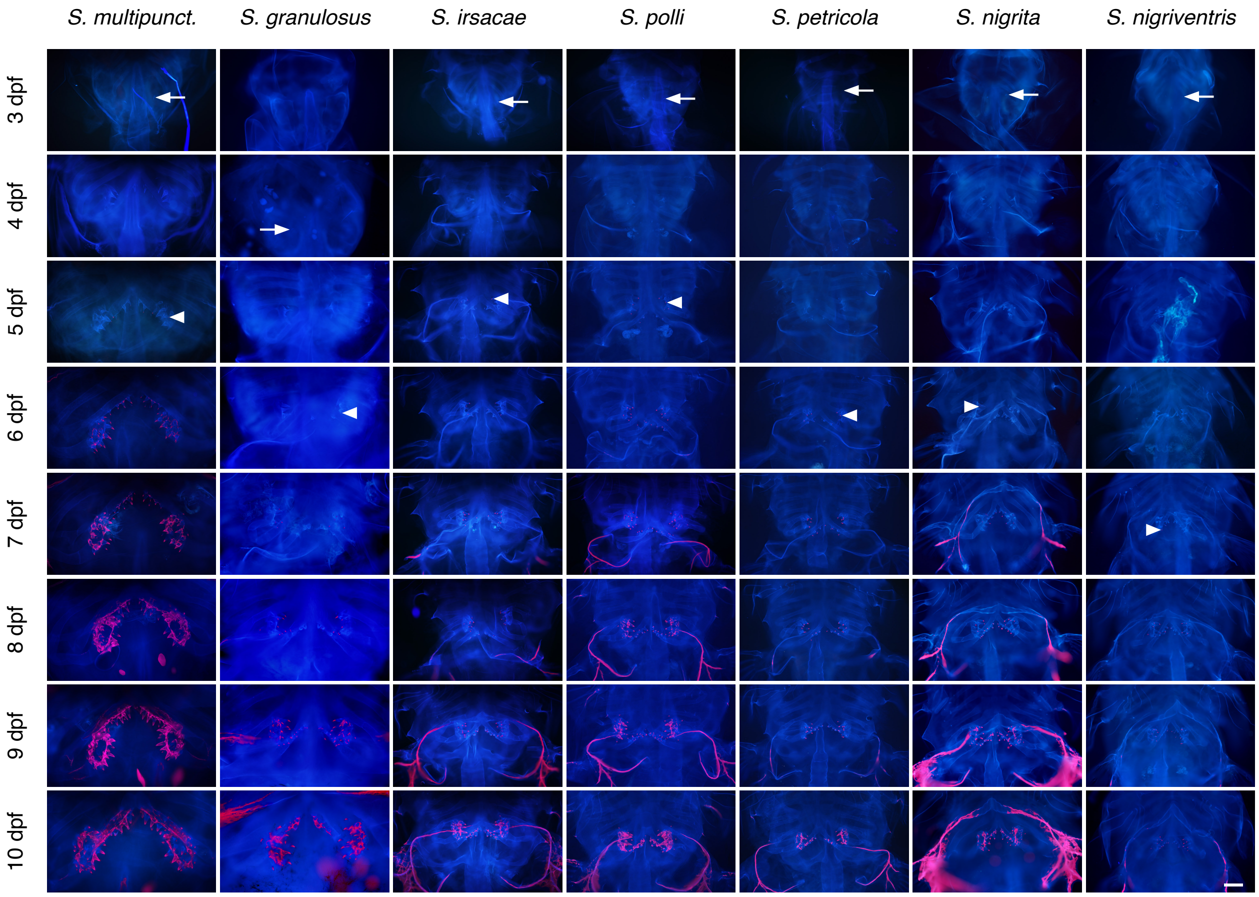

Figure S4 | Pharyngeal teeth. Arrows indicate the first appearance of the tooth, while the triangle marks the onset of tooth mineralization (shown in red in the photographs). Scale: bar = 200µm.

Table S1 | Results of an ANOVA test examining the significance of specific food categories in distinguishing between *Synodontis* species across the Lake Tanganyika radiation. Test statistics (LRT) were calculated as univariate ANOVAs from a multivariate GLM (mvabund R package) using 10,000 iterations of parametric resampling. Significance levels (P) were adjusted for multiple testing using the Holm-Bonferroni method. Significant values are indicated in bold.

Food category	Dev	P
Plant detritus	11.937	0.078
Algae	**16.224**	**0.017**
Sand	9.386	0.164
Sponges (Porifera)	**36.599**	**< 0.0001**
Zoobenthos insect	**17.797**	**0.01**
Gastropoda	9.917	0.158
Hydracarina	2.492	0.784
Caridea	5.843	0.439
Ostracoda	7.351	0.312
Copepoda	**13.646**	**0.041**
Fish scales	**16.11**	**0.017**
Terrestrial invertebrates	4.681	0.478
Fish eggs	2.846	0.784

Table S2 | Dietary contrasts within species dyads of *Synodontis* spp. The cumulative dissimilarities of the times listed account for over 80% of pairwise differences in diets, as revealed by a SIMPER analysis using Bray-Curtis dissimilarities. Average = average contribution to overall dissimilarity; sd = standard deviation of contribution; ratio = average to sd ratio; avA, avB = average abundances per species; cumsum = cumulative contribution.

Contrast A vs. B	average	sd	ratio	avA	avB	cumsum
multipunctatus - granulosus						
Fish scales	0.492	0.035	13.959	1.867	100.000	0.501
Porifera	0.155	0.192	0.812	31.067	0.000	0.660
Plant detritus	0.093	0.140	0.669	18.633	0.000	0.755
Caridea	0.069	0.126	0.545	13.733	0.000	0.825
multipunctatus - irsacae						
Porifera	0.303	0.175	1.732	31.067	84.024	0.440
Plant detritus	0.100	0.132	0.761	18.633	4.341	0.585
Caridea	0.078	0.134	0.583	13.733	2.951	0.699
Zoobenthos insect	0.061	0.101	0.603	11.967	1.244	0.787
Sand	0.049	0.117	0.424	8.233	2.390	0.859
multipunctatus - petricola						
Porifera	0.224	0.165	1.360	31.067	48.615	0.312
Plant detritus	0.125	0.125	0.997	18.633	16.667	0.485
Algae	0.125	0.141	0.889	6.300	23.538	0.659
Caridea	0.074	0.123	0.605	13.733	2.385	0.762
Zoobenthos insect	0.070	0.105	0.663	11.967	4.308	0.859
multipunctatus - polli						
Algae	0.336	0.118	2.849	6.300	73.111	0.400
Porifera	0.156	0.177	0.879	31.067	5.556	0.586
Plant detritus	0.120	0.112	1.073	18.633	16.778	0.729
Caridea	0.075	0.119	0.629	13.733	3.111	0.819
granulosus - irsacae						
Fish scales	0.501	0.004	132.617	100.000	0.000	0.501
Porifera	0.420	0.123	3.423	0.000	84.024	0.921
granulosus - petricola						
Fish scales	0.503	0.031	16.356	100.000	0.359	0.505
Porifera	0.243	0.179	1.361	0.000	48.615	0.749
Algae	0.119	0.151	0.789	0.000	23.538	0.869
granulosus - polli						
Fish scales	0.501	0.003	188.304	100.000	0.000	0.501
Algae	0.366	0.098	3.743	0.000	73.111	0.866
petricola - irsacae						
Porifera	0.230	0.172	1.338	48.615	84.024	0.442
Algae	0.122	0.144	0.845	23.538	3.488	0.676
Plant detritus	0.087	0.104	0.837	16.667	4.341	0.844
petricola - polli						
Algae	0.273	0.143	1.917	23.538	73.111	0.409
Porifera	0.229	0.169	1.354	48.615	5.556	0.752
Plant detritus	0.101	0.095	1.058	16.667	16.778	0.903
irsacae - polli						
Porifera	0.396	0.125	3.154	84.024	5.556	0.446
Algae	0.349	0.109	3.197	3.488	73.111	0.840

Table S3 | Test statistic for a GLM (two-sided) examining differences in diet composition among LT *Synodontis* species (n = 121). The model assessed the impact of LT *Synodontis* species on diet composition, using the diet content of *S. multipunctatus* as reference. Test statistics are derived from 10,000 iterations of pit-trap resampling. No adjustment for multiple testing was used.

	LR value	P
(Intercept)	490.4	0.001
S. granulosus	37.19	0.001
S. irsacae	63.96	0.001
S. petricola	32.56	0.003
S. polli	33	0.001

Table S4 | Average within-species (bold, on diagonal) and between-species Bray-Curtis dissimilarities of LT *Synodontis* species.

	S. multipunctatus	*S. granulosus*	*S. irsacae*	*S. petricola*	*S. polli*
S. multipunctatus	**0.776**	0.981	0.689	0.720	0.839
S. granulosus		**0.000**	1.000	0.996	1.000
S. irsacae			**0.259**	0.521	0.886
S. petricola				**0.570**	0.669
S. polli					**0.317**

Table S5 | Sample summary of (a) LT *Synodontis* species, and (b) LT cichlid species dissected for SIA analysis. The table includes the species name, sample size (N), as well as the mean and standard deviation (sd) for the stable isotope ratios used ($\delta^{15}N$ and $\delta^{13}C$).

Species	mean δ15N	sd δ15N	mean $\delta^{13}C$	sd $\delta^{13}C$	N
**a) LT *Synodontis*:**					
S. granulosus	8.92	0.802	-19.1	0.965	22
S. irsacae	6.31	0.300	-20.2	1.69	25
S. multipunctatus	6.23	0.522	-18.3	2.30	61
S. petricola	5.98	0.647	-18.2	2.20	42
S. polli	4.81	0.460	-12.0	3.26	48
**b) LT cichlids:**					
Altolamprologus compressiceps	5.92	0.312	-17.0	0.294	8
Boulengerochromis microlepis	7.27	0.295	-18.2	0.833	6
Eretmodus cyanostictus	4.31	0.893	-10.1	2.03	8
Lepidiolamprologus elongatus	6.60	0.182	-18.4	0.692	8
Neolamprologus fasciatus	6.45	0.267	-16.7	0.614	8
Neolamprologus sexfasciatus	6.56	0.203	-19.1	0.576	8
Paracyprichromis brieni	6.12	0.167	-21.0	0.246	8
Tropheus moorii	4.78	0.652	-14.0	0.749	8

Table S6 | Pairwise comparison of the trophic positions among LT *Synodontis* species. Trophic positions were compared using PERMANOVA, with pairwise differences calculated as Euclidean distances based on $\delta^{13}C$ and $\delta^{15}N$ values. The significance of pairwise tests was adjusted for multiple testing using the Benjamini-Hochberg procedure. Significant values are in bold.

Species pairs	Df	SumsOfSqs	F.Model	R2	P	P adjusted
**a) *Synodontis* spp.**						
S. petricola vs *S. multipunctatus*	1	2.32	0.426	0.0042	0.5376	0.5376
S. petricola vs *S. polli*	1	871.49	105.768	0.5459	0.0001	**0.0001**
S. petricola vs *S. irsacae*	1	70.83	16.132	0.1989	0.0004	**0.0005**
S. petricola vs *S. granulosus*	1	137.91	34.518	0.3576	0.0001	**0.0001**
S. multipunctatus vs *S. polli*	1	1116.79	141.584	0.5696	0.0001	**0.0001**
S. multipunctatus vs *S. irsacae*	1	66.76	13.870	0.1417	0.0008	**0.0009**
S. multipunctatus vs *S. granulosus*	1	126.45	27.938	0.2565	0.0001	**0.00019**
S. polli vs *S. irsacae*	1	1149.59	140.728	0.6647	0.0001	**0.00019**
S. polli vs *S. granulosus*	1	1001.86	125.620	0.6488	0.0001	**0.00019**
S. irsacae vs *S. granulosus*	1	97.28	42.658	0.4866	0.0001	**0.00019**

Table S7 | Results of a post-hoc pairwise analysis of least square means of clutch size within species dyads of *Synodontis* spp. SE = standard error of estimate; df = degrees of freedom. A Benjamini-Hochberg procedure was applied to account for multiple testing. Significant contrasts in bold.

Contrast	estimate	SE	df	t ratio	P value
S. multipunctatus – S. granulosus	-2.5375	0.242	71	-10.477	**<0.001**
S. multipunctatus - S. irsacae	-2.582	0.242	71	-10.66	**<0.001**
S. multipunctatus - S. polli	-2.6317	0.211	71	-12.483	**<0.001**
S. multipunctatus - S. petricola	-2.4041	0.187	71	-12.824	**<0.001**
S. multipunctatus - S. macrostigma	-2.6861	0.445	71	-6.037	**<0.001**
S. multipunctatus - S. nigrita	-4.0259	0.445	71	-9.048	**<0.001**
S. multipunctatus - S. nigriventris	-3.4873	0.372	71	-9.378	**<0.001**
S. granulosus - S. irsacae	-0.0445	0.282	71	-0.158	0.9046
S. granulosus - S. polli	-0.0941	0.256	71	-0.368	0.8691
S. granulosus - S. petricola	0.1334	0.237	71	0.563	0.7317
S. granulosus - S. macrostigma	-0.1486	0.468	71	-0.317	0.8771
S. granulosus - S. nigrita	-1.4884	0.468	71	-3.181	**0.0068**
S. granulosus - S. nigriventris	-0.9497	0.399	71	-2.38	**0.0431**
S. irsacae - S. polli	-0.0496	0.256	71	-0.194	0.9046
S. irsacae - S. petricola	0.1779	0.237	71	0.751	0.6369
S. irsacae - S. macrostigma	-0.1041	0.468	71	-0.222	0.9046
S. irsacae - S. nigrita	-1.4439	0.468	71	-3.086	**0.0075**
S. irsacae - S. nigriventris	-0.9052	0.399	71	-2.268	**0.0496**
S. polli - S. petricola	0.2276	0.205	71	1.112	0.4198
S. polli - S. macrostigma	-0.0544	0.452	71	-0.12	0.9046
S. polli - S. nigrita	-1.3942	0.452	71	-3.081	**0.0075**
S. polli - S. nigriventris	-0.8556	0.381	71	-2.247	**0.0496**
S. petricola - S. macrostigma	-0.282	0.442	71	-0.638	0.7008
S. petricola - S. nigrita	-1.6218	0.442	71	-3.669	**0.0016**
S. petricola - S. nigriventris	-1.0832	0.368	71	-2.94	**0.0103**
S. macrostigma - S. nigrita	-1.3398	0.599	71	-2.238	**0.0496**
S. macrostigma - S. nigriventris	-0.8012	0.546	71	-1.466	0.2421
S. nigrita - S. nigriventris	0.5386	0.546	71	0.986	0.4828

Table S8 | Results of a post-hoc pairwise analysis of least square means of egg size within species dyads of *Synodontis* spp. SE = standard error of estimate. A Benjamini-Hochberg procedure was applied to account for multiple testing. Significant contrasts in bold.

Contrast	estimate	SE	z ratio	P value
S. multipunctatus – S. granulosus	-0.1168	0.0437	-2.675	**0.01**
S. multipunctatus - S. irsacae	0.9171	0.0436	21.038	**<0.001**
S. multipunctatus - S. polli	0.9025	0.0436	20.704	**<0.001**
S. multipunctatus - S. petricola	1.3519	0.0436	31.014	**<0.001**
S. multipunctatus - S. macrostigma	1.245	0.0636	19.569	**<0.001**
S. multipunctatus - S. nigrita	1.3052	0.0815	16.021	**<0.001**
S. multipunctatus - S. nigriventris	1.4353	0.0815	17.619	**<0.001**
S. granulosus - S. irsacae	1.0339	0.0436	23.705	**<0.001**
S. granulosus - S. polli	1.0193	0.0436	23.371	**<0.001**
S. granulosus - S. petricola	1.4687	0.0436	33.675	**<0.001**
S. granulosus - S. macrostigma	1.3618	0.0636	21.4	**<0.001**
S. granulosus - S. nigrita	1.422	0.0815	17.453	**<0.001**
S. granulosus - S. nigriventris	1.5522	0.0815	19.05	**<0.001**
S. irsacae - S. polli	-0.0146	0.0435	-0.335	0.7377
S. irsacae - S. petricola	0.4348	0.0435	9.99	**<0.001**
S. irsacae - S. macrostigma	0.3279	0.0636	5.157	**<0.001**
S. irsacae - S. nigrita	0.3881	0.0814	4.766	**<0.001**
S. irsacae - S. nigriventris	0.5183	0.0814	6.364	**<0.001**
S. polli - S. petricola	0.4494	0.0435	10.325	**<0.001**
S. polli - S. macrostigma	0.3425	0.0636	5.387	**<0.001**
S. polli - S. nigrita	0.4027	0.0814	4.945	**<0.001**
S. polli - S. nigriventris	0.5328	0.0814	6.543	**<0.001**
S. petricola - S. macrostigma	-0.1069	0.0636	-1.682	0.1126
S. petricola - S. nigrita	-0.0467	0.0814	-0.573	0.5873
S. petricola - S. nigriventris	0.0834	0.0814	1.025	0.3422
S. macrostigma - S. nigrita	0.0603	0.0937	0.643	0.5602
S. macrostigma - S. nigriventris	0.1904	0.0937	2.032	0.0537
S. nigrita - S. nigriventris	0.1301	0.107	1.22	0.2593

Table S9 | Analysis of egg coloration along the blue-yellow (CIE B) and red-green (CIE A) axes, comparing cuckoo catfish with other LT and riverine *Synodontis* species. The results from Linear Models are presented. No adjustment for multiple testing was used.

**(a) CIE. B axis (**R^2 = 0.930)	**Species**		
Coefficient	Estimates	Conf. Int (95%)	P
(Intercept) (*S. multipunctatus*)	-5.42	-6.50 – -4.34	<0.001
S. granulosus	1.57	0.15 – 2.98	0.030
S. irsacae	3.80	2.34 – 5.26	<0.001
S. polli	2.45	0.98 – 3.91	0.001
S. petricola	4.57	3.10 – 6.03	<0.001
S. macrostigma	9.95	7.93 – 11.97	<0.001
S. nigrita	18.31	15.67 – 20.96	<0.001
S. nigriventris	9.55	6.90 – 12.19	<0.001
Analysis of Deviance Table (Type III Wald chi-squared tests)			
Intercept (d.f.)	1275.73 (1), P < 0.001		
Species (d.f.)	372.11 (7), P < 0.001		

**(b) CIE. A axis (**R^2 = 0.988)	**Species**		
Coefficient	*Estimates*	*Conf. Int (95%)*	P
(Intercept) (*S. multipunctatus*)	44.07	41.65 – 46.48	
S. granulosus	-26.46	-29.62 – -23.29	<0.001
S. irsacae	-25.96	-29.23 – -22.68	<0.001
S. polli	-20.59	-23.87 – -17.32	<0.001
S. petricola	-26.11	-29.38 – -22.84	<0.001
S. macrostigma	-19.71	-24.23 – -15.18	<0.001
S. nigrita	-31.48	-37.41 – -25.56	<0.001
S. nigriventris	-21.73	-27.65 – -15.80	<0.001
Analysis of Deviance Table (Type III Wald chi-squared tests)			
Intercept (d.f.)	96.58 (1), P < 0.001		
Species (d.f.)	289.54 (7), P < 0.001		

Table S10 | Results of a post-hoc pairwise analysis of least square means of egg adhesion within species dyads of *Synodontis* spp. SE = standard error of estimate. A Benjamini-Hochberg procedure was applied to account for multiple testing. Significant contrasts in bold.

**Contrast**	**estimate**	**SE**	**z ratio**	**P value**
S. multipunctatus – S. granulosus	0.0947	0.338	0.281	0.8726
S. multipunctatus - S. irsacae	-0.9332	0.269	-3.472	**0.0011**
S. multipunctatus - S. polli	-2.0147	0.382	-5.276	**<0.001**
S. multipunctatus - S. petricola	-3.8648	0.423	-9.126	**<0.001**
S. multipunctatus - S. macrostigma	-2.0337	0.449	-4.526	**<0.001**
S. multipunctatus - S. nigrita	-0.0532	0.58	-0.092	0.9613
S. multipunctatus - S. nigriventris	1.2604	0.733	1.719	0.1143
S. granulosus - S. irsacae	-1.028	0.343	-3	**0.0047**
S. granulosus - S. polli	-2.1094	0.438	-4.82	**<0.001**
S. granulosus - S. petricola	-3.9595	0.476	-8.313	**<0.001**
S. granulosus - S. macrostigma	-2.1285	0.498	-4.278	**0.0001**
S. granulosus - S. nigrita	-0.148	0.618	-0.24	0.8731
S. granulosus - S. nigriventris	1.1656	0.762	1.529	0.1598
S. irsacae - S. polli	-1.0814	0.384	-2.815	**0.0072**
S. irsacae - S. petricola	-2.9316	0.423	-6.937	**<0.001**
S. irsacae - S. macrostigma	-1.1005	0.451	-2.439	**0.0206**
S. irsacae - S. nigrita	0.88	0.583	1.509	0.1598
S. irsacae - S. nigriventris	2.1936	0.737	2.975	**0.0048**
S. polli - S. petricola	-1.8501	0.495	-3.738	**0.0004**
S. polli - S. macrostigma	-0.019	0.525	-0.036	0.971
S. polli - S. nigrita	1.9614	0.643	3.049	**0.0043**
S. polli - S. nigriventris	3.2751	0.788	4.157	**0.0001**
S. petricola - S. macrostigma	1.8311	0.548	3.339	**0.0017**
S. petricola - S. nigrita	3.8116	0.67	5.691	**<0.001**
S. petricola - S. nigriventris	5.1252	0.816	6.281	**<0.001**
S. macrostigma - S. nigrita	1.9805	0.686	2.889	**0.006**
S. macrostigma - S. nigriventris	3.2941	0.823	4.004	**0.0002**
S. nigrita - S. nigriventris	1.3136	0.897	1.464	0.167

Table S11 | The number of ripe ovaries and testes in four LT *Synodontis* spp. at the beginning (October) and end (March) of the rainy season, along with sample-specific size (N). The overall sample size (Ntot) for each species is also provided. The significant P-values of the sex- and species-specific chi-squared tests in gonad ripeness between the seasons are included. No adjustment for multiple testing was used.

	Females					Males					Total	
	Start		End		Chi-squared	Start		End			Ntot	Chi-squared
	ripe	N	ripe	N		ripe	N	ripe	N			
S. multipunctatus	14	20	14	14	0.200	9	10	14	14		58	0.863
S. irsacae	0	13	13	14	**<0.001**	6	14	15	15		56	**0.003**
S. polli	16	19	17	17	0.268	10	15	14	14		65	0.060
S. petricola	12	15	15	15	0.224	12	13	15	16		59	1.000

Table S12 | Notochord size at 10 days post-fertilization, with comparisons between the cuckoo catfish and other LT and riverine *Synodontis* species. The results from Linear Models on log-transformed data are presented as recalculated raw values in mm. No adjustment for multiple testing was used.

		Species	
Coefficient	*Estimates*	*Conf. Int (95%)*	*P-value*
S. multipunctatus	9.53	9.23 – 9.84	<0.001
S. granulosus	10.54	9.95 – 11.13	<0.001
S. irsacae	6.13	5.74 – 6.52	<0.001
S. polli	6.82	6.54 – 7.09	<0.001
S. petricola	5.61	5.30 – 5.91	<0.001
S. macrostigma	5.72	5.42 – 6.01	<0.001
S. nigrita	5.71	5.33 – 6.09	<0.001
S. nigriventris	9.53	9.23 – 9.84	<0.001
Analysis of Deviance Table (Type III Wald chi-squared tests)			
Intercept (d.f.)	11205.41 (1), P < 0.001		
Species (d.f.)	543.51 (6), P < 0.001		

Table S13 | Overview of *Synodontis* embryo development (ossification). The progression of ossification for a specific element is indicated in grey, while for the cuckoo catfish, it is shown in red.

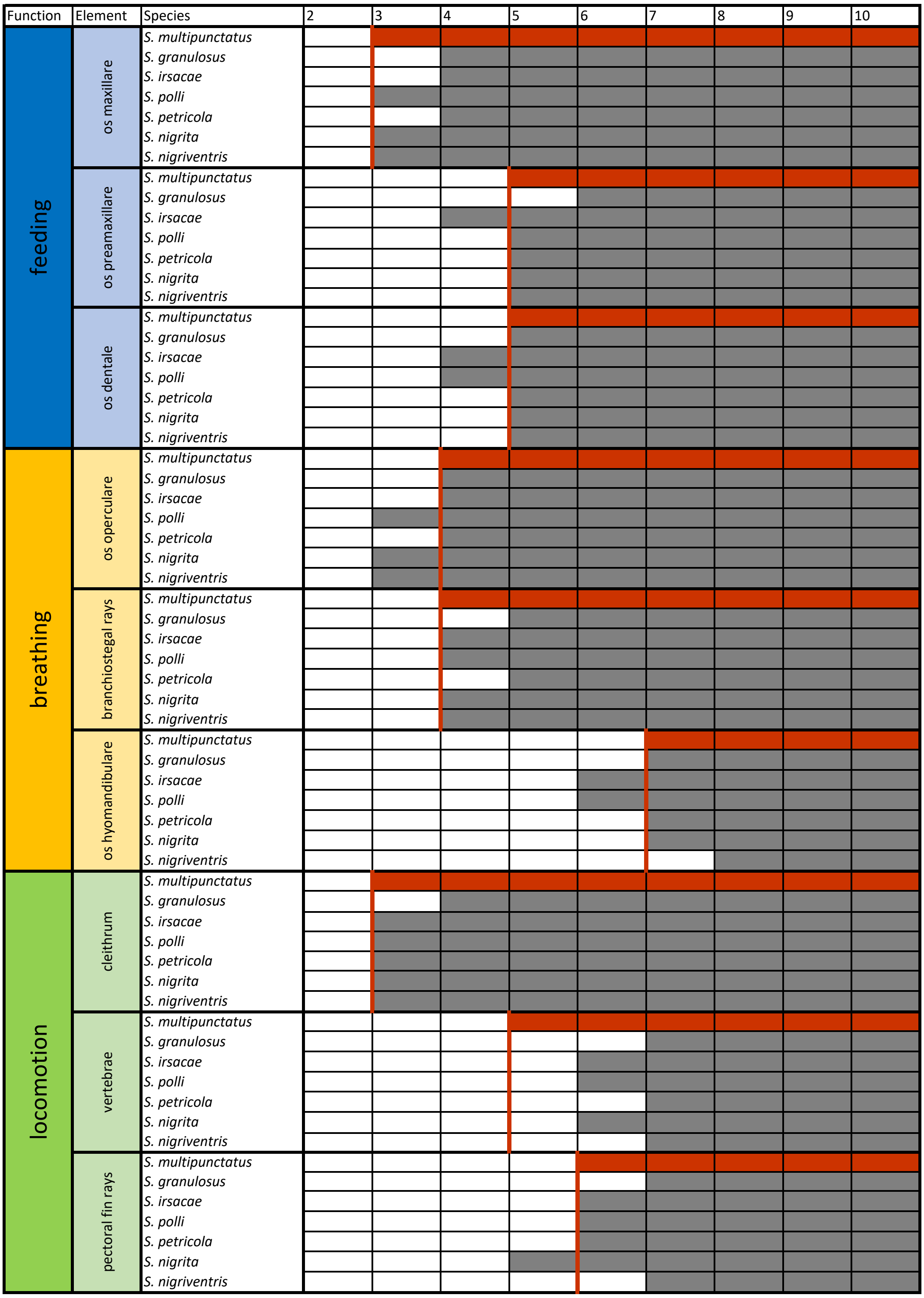

Supplement: Supplementary file 1 — Supplementary Information [file 41467_2026_71179_MOESM1_ESM.pdf]
