# Supplementary material for: The ecological and developmental foundations of brood parasitism in a catfish
Source: Nat Commun. 2026 Mar 31;17:4630. doi: 10.1038/s41467-026-71179-4 (PMC13199399; doi:10.1038/s41467-026-71179-4)
Supplement: Supplementary file 3 — Reporting Summary [file 41467_2026_71179_MOESM3_ESM.pdf]

nature portfolio

Corresponding author(s): Martin Reichard

Last updated by author(s): 16/2/26

Reporting Summary

Nature Portfolio wishes to improve the reproducibility of the work that we publish. This form provides structure for consistency and transparency in reporting. For further information on Nature Portfolio policies, see our Editorial Policies and the Editorial Policy Checklist.

Statistics

For all statistical analyses, confirm that the following items are present in the figure legend, table legend, main text, or Methods section.

n/a	Confirmed	
[ ]	[x]	The exact sample size (*n*) for each experimental group/condition, given as a discrete number and unit of measurement
[ ]	[x]	A statement on whether measurements were taken from distinct samples or whether the same sample was measured repeatedly
[ ]	[x]	The statistical test(s) used AND whether they are one- or two-sided<br>*Only common tests should be described solely by name; describe more complex techniques in the Methods section.*
[ ]	[x]	A description of all covariates tested
[ ]	[x]	A description of any assumptions or corrections, such as tests of normality and adjustment for multiple comparisons
[ ]	[x]	A full description of the statistical parameters including central tendency (e.g. means) or other basic estimates (e.g. regression coefficient) AND variation (e.g. standard deviation) or associated estimates of uncertainty (e.g. confidence intervals)
[ ]	[x]	For null hypothesis testing, the test statistic (e.g. *F*, *t*, *r*) with confidence intervals, effect sizes, degrees of freedom and *P* value noted<br>*Give P values as exact values whenever suitable.*
[x]	[ ]	For Bayesian analysis, information on the choice of priors and Markov chain Monte Carlo settings
[ ]	[x]	For hierarchical and complex designs, identification of the appropriate level for tests and full reporting of outcomes
[x]	[ ]	Estimates of effect sizes (e.g. Cohen's *d*, Pearson's *r*), indicating how they were calculated

Our web collection on statistics for biologists contains articles on many of the points above.

Software and code

Policy information about availability of computer code

Data collection	No software was used.
Data analysis	R 4.2.3 Details on specific libraries are in the Methods section.<br>glmmTMB<br>DHARMa<br>adonis<br>vegan<br>mvabund<br>IQ-TREE v.2.0.7<br>emmeans<br>phytools<br>geiger

For manuscripts utilizing custom algorithms or software that are central to the research but not yet described in published literature, software must be made available to editors and reviewers. We strongly encourage code deposition in a community repository (e.g. GitHub). See the Nature Portfolio guidelines for submitting code & software for further information.

Data

Policy information about availability of data

All manuscripts must include a data availability statement. This statement should provide the following information, where applicable:

- Accession codes, unique identifiers, or web links for publicly available datasets
- A description of any restrictions on data availability
- For clinical datasets or third party data, please ensure that the statement adheres to our policy

Data associated with this manuscript are available in the supplementary materials and at Figshare (doi: 10.6084/m9.figshare.28646141).

Research involving human participants, their data, or biological material

Policy information about studies with human participants or human data. See also policy information about sex, gender (identity/presentation), and sexual orientation and race, ethnicity and racism.

Reporting on sex and gender	*Use the terms sex (biological attribute) and gender (shaped by social and cultural circumstances) carefully in order to avoid confusing both terms. Indicate if findings apply to only one sex or gender; describe whether sex and gender were considered in study design; whether sex and/or gender was determined based on self-reporting or assigned and methods used.*<br>*Provide in the source data disaggregated sex and gender data, where this information has been collected, and if consent has been obtained for sharing of individual-level data; provide overall numbers in this Reporting Summary. Please state if this information has not been collected.*<br>*Report sex- and gender-based analyses where performed, justify reasons for lack of sex- and gender-based analysis.*
Reporting on race, ethnicity, or other socially relevant groupings	*Please specify the socially constructed or socially relevant categorization variable(s) used in your manuscript and explain why they were used. Please note that such variables should not be used as proxies for other socially constructed/relevant variables (for example, race or ethnicity should not be used as a proxy for socioeconomic status).*<br>*Provide clear definitions of the relevant terms used, how they were provided (by the participants/respondents, the researchers, or third parties), and the method(s) used to classify people into the different categories (e.g. self-report, census or administrative data, social media data, etc.)*<br>*Please provide details about how you controlled for confounding variables in your analyses.*
Population characteristics	*Describe the covariate-relevant population characteristics of the human research participants (e.g. age, genotypic information, past and current diagnosis and treatment categories). If you filled out the behavioural & social sciences study design questions and have nothing to add here, write "See above."*
Recruitment	*Describe how participants were recruited. Outline any potential self-selection bias or other biases that may be present and how these are likely to impact results.*
Ethics oversight	*Identify the organization(s) that approved the study protocol.*

Note that full information on the approval of the study protocol must also be provided in the manuscript.

Field-specific reporting

Please select the one below that is the best fit for your research. If you are not sure, read the appropriate sections before making your selection.

☐ Life sciences ☐ Behavioural & social sciences ☒ Ecological, evolutionary & environmental sciences

For a reference copy of the document with all sections, see nature.com/documents/nr-reporting-summary-flat.pdf

Ecological, evolutionary & environmental sciences study design

All studies must disclose on these points even when the disclosure is negative.

Study description	New datasets on diet, stable isotopic ratios, reproductive and dvelopmental traits from samples collected for this study in the field and laboratory.
Research sample	Adult Synodontis and cichlids were collected during nine field expeditions to Zambia and Tanzania between 2019 and 2024. Samples of all species Lake Tanganyika Synodontis catfish were targeted. Additionally, developmnetal series of Synodontis species from LT and adjacent rivers from captive animals.
Sampling strategy	Fish were collected during Scuba dives using hand nets, stop nets, and baited minnow traps and brought to the surface for further processing. Additional samples (of deepwater S. granulosus) were obtained from deep-water gill nets deployed by local fishermen. Fish were typically processed within the first two hours after collection. They were euthanized with an overdose of clove oil, and a piece of fin was stored in 96% ethanol for DNA barcoding and further genetic analyses. The fish were measured, weighed, labelled, fixed in 6% formaldehyde, and subsequently transferred to 70% ethanol for long-term storage. A piece of muscle from the fish flank (dorsolateral side) was taken and stored in ethanol for the analysis of stable isotope ratios. Vouchers are stored at the Natural History Museum in Vienna, and collections are held at the Institute of Vertebrate Biology, Czech Academy of Sciences. Morphological

	identifications were confirmed by DNA barcoding using the cytochrome c oxidase I gene.<br>A diet analysis was conducted on samples collected from the southern tip of the lake in Zambia, specifically at Mpulungu (Chituta Bay, Kalambo Lodge, Mutondwe Island, Mpulungu) and Ndole Bay (Chimba Rocks, Mpende Fisheries, Katete, and Cape Kachese), where all examined fish species co-occur locally. All primary sample data are georeferenced.<br>A comparison of egg traits (size, adhesiveness, coloration) and developmental traits among five LT species and three riverine species of Synodontis was conducted on eggs obtained following hormonal stimulation of fish.
Data collection	All data were collected during nine field expeditions to Zambia and Tanzania between 2019 and 2024 and in the labortory between 2021 and 2025. Field data collection: MR, HZ, RB, MP, GE, VB, LK, SK. Laboratory data collection: HZ, RB, TS, MP, GE, KP, JŽ, LK, ID
Timing and spatial scale	All collections were done between 2019 and 2025, during one or two sampling trips each year. Entire Tanzanian and Zambian region of LT was targeted. Samples are georeferenced. Most field data come from Zambian region of the lake (diet, stable isotopes).
Data exclusions	Whenever data were excluded (diet anaysis: empty alimentary tracts or only digested and unidentifiable food), it is indicated in the methods.
Reproducibility	Experiments were not replicated. All primary data are stored nd accessible at Figshare. For stable isotopic data, every 5th sample was analysed twice.
Randomization	The work did not necessitate random assignment of individuals to treatment groups.
Blinding	Blinding was not applied.

Did the study involve field work? [x] Yes [ ] No

Field work, collection and transport

Field conditions	All samples collected in the field were preserved in formalin or ethanol prior to transport. Environmental data are not relevant for the study.
Location	Exact GPS location are provided for each sample in the datasheet deposited at Figshare and accessible to reviewers and readers.<br>Most samples originate from -8.6237318, 31.20110554 (site Kalambo Falls Lodge, Mpulungu, Zambia)
Access & import/export	In Zambia, research was conducted under research permit K-4335/18 KA/K.48/18 and individual study permits of MR, HZ, RB, MP, VB and SK. In Tanzania, samples were collected under research permits from COSTECH (2022-204-NA-2022-228) and TAFIRI (TAFIRI/HQ/RES.CLEARANCE/82). Import of samples to EU has been approved by Czech State Veterinary Administration (Zambia: SVS/2024/064505-G, Tanzania: SVS/2024/064505-G).
Disturbance	Only few individuals were collected from each population. Scuba diving, baited metal traps and hand net collection were applied which minimize any disturbance to non-target organisms. No study species is under conservation risk.

Reporting for specific materials, systems and methods

We require information from authors about some types of materials, experimental systems and methods used in many studies. Here, indicate whether each material, system or method listed is relevant to your study. If you are not sure if a list item applies to your research, read the appropriate section before selecting a response.

Materials & experimental systems

n/a	Involved in the study	
[x]	[ ]	Antibodies
[x]	[ ]	Eukaryotic cell lines
[x]	[ ]	Palaeontology and archaeology
[ ]	[x]	Animals and other organisms
[x]	[ ]	Clinical data
[x]	[ ]	Dual use research of concern
[x]	[ ]	Plants

Methods

n/a	Involved in the study	
[x]	[ ]	ChIP-seq
[x]	[ ]	Flow cytometry
[x]	[ ]	MRI-based neuroimaging

Animals and other research organisms

Policy information about studies involving animals; ARRIVE guidelines recommended for reporting animal research, and Sex and Gender in Research

Laboratory animals	Synodontis multipunctatus<br>S. granulosus<br>S. irsacae<br>S. polli

	S. petricola<br>S. macrostigma<br>S. nigrita<br>S. nigriventris
Wild animals	Synodontis multipunctatus<br>S. granulosus<br>S. irsacae<br>S. polli<br>S. petricola<br><br>Altolamprologus compressiceps<br>Boulengerochromis microlepis<br>Eretmodus cyanostictus<br>Lepidiolamprologus elongatus<br>Neolamprologus fasciatus<br>Neolamprologus sexfasciatus<br>Paracyprichromis brieni<br>Tropheus moorii
Reporting on sex	Whenever appropriate, sexes were analysed separately (investment to gonads). For the diet and stable isotope data, sexes were pooled and were equally represented.
Field-collected samples	Adult catfish were housed in 350 L tanks (1.30 m long, 0.60 m wide, 0.45 m deep), with a sand substrate (grain size 2-4 mm) and two ceramic shelters. Water temperature ranged between 26 and 28 °C. Water quality was maintained by internal filters, continuous aeration and partial weekly water changes (one third of water volume). Photoperiod was 13 h light and 11 h dark.<br>These fish were commercially imported and later used for artificial reproduction to obtain eggs and embryos.
Ethics oversight	Research adhered to all national and institutional animal care and use guidelines (permit No. CZ62760203 from Ministry of Agriculture.

Note that full information on the approval of the study protocol must also be provided in the manuscript.

Plants

Seed stocks	*Report on the source of all seed stocks or other plant material used. If applicable, state the seed stock centre and catalogue number. If plant specimens were collected from the field, describe the collection location, date and sampling procedures.*
Novel plant genotypes	*Describe the methods by which all novel plant genotypes were produced. This includes those generated by transgenic approaches, gene editing, chemical/radiation-based mutagenesis and hybridization. For transgenic lines, describe the transformation method, the number of independent lines analyzed and the generation upon which experiments were performed. For gene-edited lines, describe the editor used, the endogenous sequence targeted for editing, the targeting guide RNA sequence (if applicable) and how the editor was applied.*
Authentication	*Describe any authentication procedures for each seed stock used or novel genotype generated. Describe any experiments used to assess the effect of a mutation and, where applicable, how potential secondary effects (e.g. second site T-DNA insertions, mosiacism, off-target gene editing) were examined.*
